# In vitro tools for orally inhaled drug products—state of the art for their application in pharmaceutical research and industry and regulatory challenges

**DOI:** 10.1007/s44164-021-00003-8

**Published:** 2021-12-21

**Authors:** Julia Katharina Metz, Marius Hittinger, Claus-Michael Lehr

**Affiliations:** 1 Department of Drug Delivery, PharmBioTec Research & Development GmbH, 66123 Saarbrücken, Germany; 2 Department of Pharmacy, Saarland University, 66123 Saarbrücken, Germany; 3 Helmholtz Institute for Pharmaceutical Research Saarland (HIPS), Helmholtz Center for Infection Research (HZI), 66123 Saarbrücken, Germany

**Keywords:** 3R Principle, OECD guidelines, Orally inhaled drug products

## Abstract

The drug development process is a lengthy and expensive challenge for all involved players. Experience with the COVID-19 pandemic underlines the need for a rapid and effective approval for treatment options. As essential prerequisites for successful drug approval, a combination of high-quality studies and reliable research must be included. To this day, mainly in vivo data are requested and collected for assessing safety and efficacy and are therefore decisive for the pre-clinical evaluation of the respective drug. This review aims to summarize the current state of the art for safety and efficacy studies in pharmaceutical research and industry to address the relevant regulatory challenges and to provide an outlook on implementing more in vitro methods as alternative to animal testing. While the public demand for alternative methods is becoming louder, first examples have meanwhile found acceptance in relevant guidelines, e.g. the OECD guidelines for skin sensitizer. Besides ethically driven developments, also the rather low throughput and relatively high costs of animal experiments are forcing the industry towards the implementation of alternative methods. In this context, the development of orally inhaled drug products is particularly challenging due to the complexity of the lung as biological barrier and route of administration. The replacement of animal experiments with focus on the lungs requires special designed tools to achieve predictive data. New in vitro test systems of increasing complexity are presented in this review. Limits and advantages are discussed to provide some perspective for a future in vitro testing strategy for orally inhaled drug products.

**Graphical abstract:**

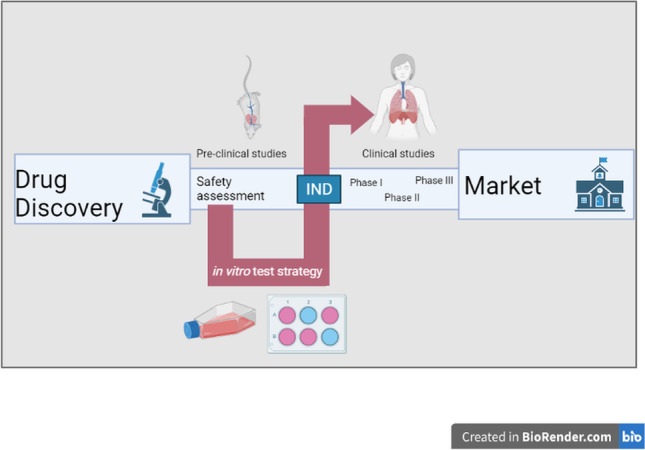

## Introduction

### Brief introduction

The directive 2010/63/EU of the European parliament and of the council underlines that the final goal is ‘full replacement of procedures on live animals for scientific and educational purposes’ [1]. But it also states that for safety and efficacy, a ‘regulatory testing’ is necessary from which some requirements can only be fulfilled by animal experiments [1]. An example of a full replacement is the application of in vitro safety assessment of chemicals in the cosmetics industry [2]. The regulation (EC) No. 1223/2009 of the European Parliament and of the Council, which came into force on November 30, 2009, states that animal experiments for testing finished cosmetics are forbidden by law [3, 4]. This regulation underlines the political pressure on developing new alternatives and on the other side the ability of alternatives for predicting human data. For pulmonary drug development, which is nothing else than inhaling chemicals with an intended safe use and a specific efficacy, several in vitro methods are available but animal experiments are still regulatory required. Within this review, we will summarize and discuss the aspects of chemical registration and drug approval in the context of a potential animal testing free drug development.

### The price we pay

Animal experiments are quite expensive, in some cases ethically unacceptable, and they often produce results that are not valid enough to predict potential safety issues in humans [5, 6]. In 2011, 1.3 billion € was spent on animal experiments for the safety assessment of chemicals in total, whereby the acute inhalation toxicity is calculated with 13.85 million € and sub-chronic toxicity studies with 61.95 million € [7]. In the first report for the ‘EU statistical data on the use of animals for scientific purposes’ in 1994, 11,790,485 animal experiments were conducted in the European Union [8]. The total number of animals was decreased to 9,390,000 in 2017, which is communicated in the newest report from the European Commission in 2020 [9].

Next to these considerable costs, the predictivity of animal experiments is called into question by numerous studies. Only 71% of two-species studies can predict the human toxicity studies [10], resulting in a 89% failure rate of new drugs after human clinical trials [5]. Reasons for this low predictivity are physiological differences between the species that lead to, among other things, diverse metabolism functions, varied microbiome constitutions, altered gene expression profiles and differently expressed disease phenotypes [10].

In response to these limitations of animal experiments, the demand for suitable in vitro methods is increasing. Goh et al. calculated all in vitro assays performed in the main three fields genotoxicity, safety pharmacology and ADME of pre-clinical studies from three pharma companies and three contract research organizations (CRO) in the years 1980 to 2013 with the result that the percentage of performed in vitro assays in the pharma industry has increased by a remarkable 20% since 2012 [11]. The actual market of in vitro toxicology testing was estimated at $ 22.7 billion in 2020 and extrapolations demonstrate that the market size of the in vitro toxicology testing will be increased up to 11.4% in 2028 [12]. This increasing number of in vitro assays used for scientific purposes can be related to a slight reduction in animal testing from 1990 until 2017 even if there are strict regulatory requirements.

## Regulatory requirements for safety assessment of chemicals and pharmaceuticals

The European Union’s REACH regulation ((EC) 1907/2006) legally regulates the market and safety of chemicals since 2007. According to the slogan ‘no data, no market’, chemical manufacturers must provide all necessary data for a detailed safety evaluation for the product that they want to release to the market [13]. The best way for a uniform evaluation of the product is to adhere to certain guidelines. Relating thereto, the OECD, an international association with 36 member states aiming for the reduction of economic, political and environmental complications through standardized procedures, is responsible for the development of such guidelines [14]. The internationally accepted OECD guidelines include the (bio)safety assessment of chemicals and the protection of the environment and human health. This collection of guidelines for the testing of chemicals is divided into 5 sections: (1) physical–chemical properties; (2) effects on biotic systems; (3) environmental fate and behaviour; (4) health effects and (5) other test guidelines [15]. In the 4^th^ section of guidelines, 80 OECD regulations describe suitable methods for identifying potential health risks of the substances under test. For example, the (acute) toxicity, genotoxicity, neurotoxicity, sensitization and corrosion effects of the test substance are addressed in in vitro and in vivo studies depending on the organ affected [16]. After the aforementioned health risks for human and environment can be assessed, the manufacturer has to apply to the ECHA (European Chemical Agency) for registration, depending on the production volume per year. If approved, the chemical can be placed on the market [17].

In contrast to chemicals, drugs have to demonstrate safety and efficacy for human application. As soon as a potent drug candidate has been identified, several development phases must be passed through. Often, the development process starts with a chemical optimization of the active compound, which serves to improve the physiochemical and biological characteristics to achieve an increased efficacy (lead compound optimization) [18]. In the pre-clinical phase, the targeted compound is tested mainly in short- and long-term animal studies for toxicology and disease-modifying effects [19].

In principle, the pre-clinical testing for orally inhaled drug products is the same as for other routes of applications as parenteral or oral. The manufacturer must provide data which include especially in vivo repeat-dose toxicology (up to 9 months) studies. These studies are performed to exclude toxic effects directly in the lung tissue and avoid systemic toxicity. This will include respiratory tract malfunctions, e.g. larynx and nasal cavity irritancy. As examples, common drugs for inhalation, e.g. salbutamol, fluticasone or tobramycin, were tested in this way [20, 21]. All these studies are performed in vivo, it is recommended to use one rodent and one non-rodent species to evaluate the safety of the drug formulation [21]. A first goal of this testing is the identification of the no-observed-adverse-effect levels (NOAELs) which specify the initial dose for starting the clinical phase I [19, 22].

**Fig. 1:**
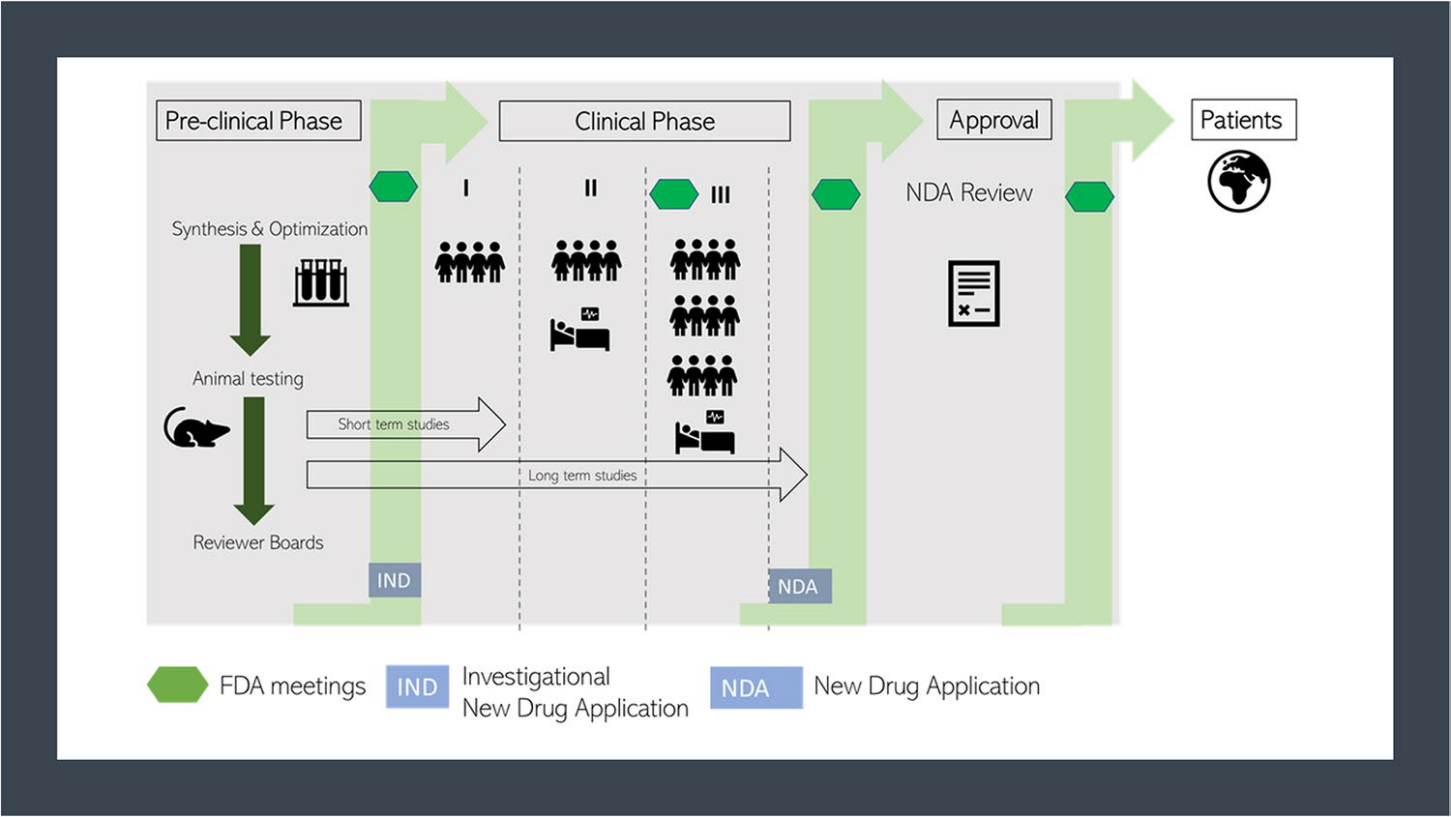
Overview of the drug development and approval process of new drugs by the FDA. An investigational new drug (IND) undergoes during their development various phases of pre-clinical research and clinical studies. After a successful synthesis and purification, the IND will be tested in the pre-clinical phase for their efficacy and safety by inter alia short- and long-term animal studies followed by an official IND submission. Passing the three phases of clinical studies, the advisory committees decide for an approval of the drug product [22]. Reprinted from: ‘New Drug Development and Review Process’, copyright © 2020 FDA homepage, https://www.fda.gov/drugs/cder-small-business-industry-assistance-sbia/new-drug-development-and-review-process, accessed 01/03/2021

If there are no safety concerns after the pre-clinical phase, the developing company can submit an approval for investigatory new drug (IND) to the US Food and Drug Administration (FDA) or clinical trial application (CTA) by the European Medicines Agency (EMA) [23]. After authorisation has been obtained from the IND or CTA, respectively, the clinical trials start. With an increasing number of participants in the clinical phase, the safety and efficacy of the IND are evaluated in humans until the data situation allows for the preparation of a new drug application (NDA) which is then sent to the FDA or EMA. An independent advisory committee of the agency will discuss the results of the NDA and decide if the drug promises good treatment options for the patients [22, 24]. During the final drug approval phase in Europe, the different national committees can sometimes also be involved in the ultimate approval decision [24]. Occasionally, the regular drug approval process can be accelerated due to an emergency, like during the COVID-19 pandemic, where the BioNTech/Pfizer vaccine received approval after 10 months, compared to an average of 15 years or longer in the regular approval process [25]. Moreover, drug approval processes can be changed when it comes to the approval of orphan drugs including a reduced clinical phase II in 4 years compared to 6 years for standard approvals [24, 26, 27]. Figure 1 summarizes the drug development process which must be passed for a final FDA approval.

As mentioned above a key element during the drug development process is the IND application, which is crucial for the start of clinical studies in humans. It stands to reason that potential safety issues of a new drug should be clarified in advance. This is done in IND enabling studies which cover the pre-clinical safety assessment by a complete description of the pharmacodynamics and kinetics (ADME properties), safety pharmacology, reproductive and developmental toxicity, and genotoxicity studies [28]. The FDA provides various forms which must be submitted for IND application. One of the most important forms is the FDA 1571 *Title 21, Code of Federal Regulations (CFR) Part 312* including a detailed description of the data situation in the pre-clinical evaluation [28–31]. To ensure FDA acceptance of the data integrated in an IND application, internationally accepted good laboratory and manufacturing practice (GLP/GMP) and quality guidelines from the ‘Organisation for Economic Co-operation and Development’ (OECD) and the ‘International Council for Harmonisation of Technical Requirements for Pharmaceuticals for Human Use’ (ICH) should be met [32–34].

The ICH, a consortium of key players from regulatory authorities and pharmaceutical industries, has established standards on quality, safety and efficacy, and also provides the multidisciplinary guidelines, all of which help to unify the international drug development process [35]. These guidelines ensure that adequate data quality can be guaranteed, which makes a successful drug approval more likely. The ICH guidance document M3 (R2), for example, describes in detail the required data for the pre-clinical evaluation of pharmaceuticals to support subsequent clinical trials [36, 37]. Safety assessments do not only play an essential role during drug approval, they are also important for the registration and market authorization of chemicals and, consequently, their commercial distribution and industrial processing [38].

In order to adequately comply with the law and, thus, ensure safety for humans and the environment, animal experiments are still required [39] and are essential from regulatory perspective (e.g. ECHA) as no adequate and valid alternatives are available [40].

## Changing the perspective: replacing animal experiments by predicting human data

It is an obvious strategy that animal experiments might be replaced most efficiently by methods leading to the same results as animal experiments do. This concept will transfer all disadvantages such as low predictivity of human data as well. This low predictivity is mainly localized in efficacy and not safety-related questions. From this perspective and with focus on pulmonary drug development, a particular understanding of relevant lung diseases is necessary before developing adequate in vitro tools.

According to the World Health Organization (WHO), chronic obstructive pulmonary disease and lower respiratory infections occupy the 3^rd^ and 4^th^ places, respectively, of the most common causes of death worldwide [41]. The lethality of patients suffering from lung diseases will rise in the future due to the COVID-19 pandemic [42]. The most relevant lung disorders are divided into four main categories: (1) acute lung diseases, e.g. pneumonia and influenza; (2) chronic inflammatory diseases, e.g. chronic obstructive pulmonary disease (COPD) and asthma; (3) occupational lung disorders, such as various forms of lung fibrosis; and (4) parenchymal lung diseases, mostly related to immune disorders [43].

Chronic lung complications are a cause of underlying diseases mostly because of previous acute lung failures such as infections [44]. Chronic lung conditions such as asthma and COPD are mainly triggered by allergies and an increased inflammatory response due to air contaminations, e.g. cigarette smoke and noxious gases [45, 46]. Acute lung diseases such as pneumonia are caused by lower respiratory tract infections of bacteria or viruses [47], resulting mostly in acute lung injury (ALI) or acute respiratory distress syndrome (ARDS) depending on the symptom severity [48–50]. This diversity of lung diseases leads to an enormous range of treatment options. For example, in the ‘Guidelines for the management of adult lower respiratory tract infections’, the European Respiratory Society (ERS) has listed various antibiotic treatment options, e.g. amoxicillin or tetracyclines, depending on the degree and type of infection, and recommended that vaccination is suitable for risk prevention [51]. The Global Initiative for Chronic Obstructive Lung Disease (GOLD) compiled a list with medical formulations and typical doses for the pharmacological treatment of COPD. Next to inhalable corticosteroids, anticholinergics, beta_2_-agonists and various combinations can be applied via metered dose inhaler (MDI) or dry powder inhaler (DPI) to COPD patients [52, 53].

Apart from these classic pharmacological interventions, innovative therapies, especially for the cure of ARDS due to the spread of COVID-19, are on their way for regulatory approval. At the end of 2019, Silva et al. discussed that personalized medicine can offer targeted therapies including coordinated treatments for the individual patient’s biochemical and physiological reconstitution during ARDS [54]. In addition, cell therapies with (embryonic) stem cells promise in vivo a reconstitution of damaged lung epithelial cells during ALI/ARDS, whereas innovative gene therapies can influence protein expression responsible for the regulation of inflammatory signalling pathways [55]. A lot of active pharmaceutical ingredients (API) with potential ARDS treatment options are currently in the pre-clinical phase of drug development. Examples of such APIs are common anti-coagulants with effects against vascular dysfunction, immunomodulatory pleiotropic and pathway-specific consequences (e.g. elafin and anti-INF-γ therapies), and anti-viral agents such as remdesivir and favipiravir which have been heavily promoted in their development during the COVID-19 pandemic in 2020 [56]. A further example for an innovative ARDS treatment is the hormone therapy based on PEG-adrenomedullin submitted as an orphan drug approval by Bayer AG Pharmaceuticals with indication for ARDS in clinical phase II [57, 58]. Next to the local treatment of lung diseases, the inhalative application route, e.g. for insulin therapy or against hypertension, has various advantages, as a faster treatment, higher efficacy and a reduced systemic side effect than other ways of administration [59]. These drug developments and approvals are essential for new therapeutic options to treat the above-mentioned lung diseases. Nevertheless, only a few drug candidates pass the long and financially risky development process to eventually achieve official approval [60]. The reasons for the high failure rate and how in vitro models can be advantageously involved in the drug development process, also considering regulatory aspects, are described in the following section.

## Validation is the challenge to face

Significant progress was achieved by replacing animal experiments with focus on skin data, such as the skin sensitization test (No. 442 C-E) and the in vitro skin irritation test (No. 439), and also many in vitro genotoxicity tests, as the mammalian cell micronucleus test (No. 487). However, no in vitro method for the inhalative safety assessment is yet recommended by the OECD. Consequently, the required data must still be generated by animal experiments. The official guidelines for the applied in vivo methods are No. 403, 433, and 436, reporting the acute toxicity, and the guidelines no. 412 and 413, analysing the sub-acute and sub-chronical effects to the lung after substance exposure (Table 1).

**Table 1:** OECD guidelines for the inhalative safety assessment of chemicals performed by in vivo studies. *TG No.*, test guideline number; *LC50*, lethal concentration; *GHS*, Globally Harmonized System; *BMC*, benchmark concentration; *NOAEC*, no observed adverse effect concentration; *LOAEC*, lowest observed adverse effect concentration

TG No	Indication	Aim	Instruments	Reference
Acute toxicity				
403	Acute inhalation Toxicity, 4 h	LC50	Inhalation chamber (nose-only, whole-body)	[43]
433	Fixed concentration Procedure, 4 h	Evident toxicity	Inhalation chamber (head/nose-only, whole-body)	[44]
436	Acute toxic class (ATC) method, 4 h	Fixed concentrations, step wise to GHS	Inhalation chamber (head-only, nose-only, snout-only)	[45]
Sub-acute toxicity				
412	Repeated dose Inhalation toxicity, 28-day study	Quantitative risk Assessments BMC, NOAEC, LOAEC	Inhalation chamber (head-only, nose-only, snout-only)	[46]
Sub-chronic toxicity				
413	Sub-chronic Inhalation toxicity, 90-day study	Quantitative risk Assessments BMC, NOAEC, LOAEC	Inhalation chamber (head-only, nose-only, snout-only)	[47]

On December 12, 2013, the European Union Reference Laboratory for Alternatives to Animal Testing (EURL ECVAM) recommended the Direct Peptide Reactivity Assay (DPRA) testing to evaluate potential skin sensitizers [61]. The methods for in vitro evaluation of skin sensitizer were expanded to the cell-based assays KeratinoSens™, LuSens, h-CLAT, (m)MUSST showing a better predictivity to human data [62]. Urbisch et al. show that in silico methods as the QSAR (quantitative structure–activity relationship) Toolbox and TIMES (tissue metabolism simulator) were more reliable to identify skin sensitizer in comparison to the in vivo LLNA (local lymph node assay) [63]. In 2018, the ECHA recommended the in vitro skin sensitizing methods within the safety assessment to meet the REACH regulation according to the OECD guidelines 442 C-E [64]. In addition, other in vitro test guidelines are officially accepted, e.g. test no. 498: in vitro phototoxicity assay, test no. 490: in vitro mammalian cell gene mutation assay, and test no. 473: in vitro mammalian chromosomal aberration test [65]. A complete list of all validated in vitro test methods is given by the EURL ECVAM on their web page [66]. Although lot of in vitro assays, for examining the skin sensitizer, eye corrosion and gene mutation, were successfully established in guidelines, no officially OECD test guidelines are available for an inhalation toxicity assessment of chemicals. To guide developers of in vitro methods to a potential validation and approval of their in vitro assays, the OECD published the ‘Guidance Document on Good In Vitro Method Practices (GIVIMP)’ in 2018. Based on GMP/GLP system, all important points during the establishment and application of an in vitro method are considered in this GIVIMP guideline. Starting with the qualification of the suppliers of the lung cell cultures (e.g. ATCC, DSMZ), to the necessary quality assurance (QA) and quality control (QC) during validation and application of the method, is described in detail. Further rules, such as the equipment of the facilities, as laminar flow benches, needed material (e.g. cell culture vessels, pipettes) and reagents (e.g. cell culture media, chemicals, supplements), must also be audited and must comply with the GMP/GLP system. A suitable reference/control representing the applicability of the method must be selected within the assay, for example, in a cell viability assay, the unexposed control with buffer and a negative control with a cell destroying detergent. The results must be sufficiently documented and archived so that anyone can check the results for transparency, reproducibility, robustness and accuracy at any time [67]. There is currently no officially recognized in vitro method that fulfils the requirements just mentioned. But many systems are promising, are well-established and are in the starting blocks for validation. They differ in their complexity from simple monolayer 2D cultures to mixed cultures (co-cultures), 3D cultures and overly complex co-cultures with more than three cell types. The costs of these test systems increase but the physiological relevance grows as well. But none of the test systems is validated under permitted guidelines recommended from the OECD or ICH [68]. A complete validation of an applicable in vitro method implies a successful transfer from research to industry [68, 69]. The coordination of this validation process is carried out by the US Interagency Coordinating Committee on the Validation of Alternative Methods (ICCVAM) [70] and, for the European Union, by the EURL ECVAM [71]. In the ECVAM status report from 2019, next to various in vitro assays, e.g. CATMoS (in silico models of acute oral systemic toxicity), in vitro cytotoxicity test 3T3 Neutral Red Uptake and the MELN® human estrogen receptor transcriptional activation assay, only two alternatives for respiratory in vitro alternatives are submitted for validation: (1) the in vitro system ALIsens, which should replace the respiratory local lymph node (LLNA) assay, and (2) undergoing validation at the time of publication, the EpiAirway™ system, a reconstructed human lung epithelium for detecting acute inhalation toxicity studies [72].

## The potential power of in vitro assays for chemical and pharmaceutical industry

**Fig. 2:**
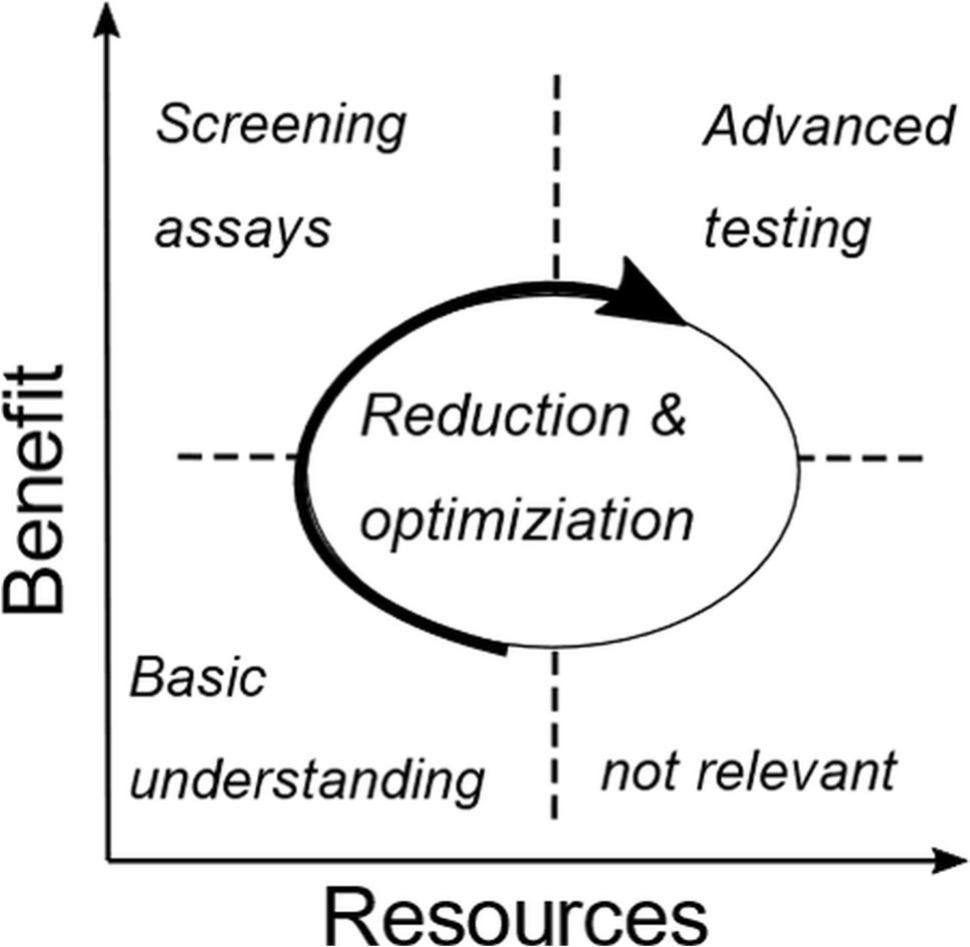
Benefits versus resources during an in vitro method development. Starting from the basic understanding to screening assays ending to advanced testing, the reduction of complexity increases to support the optimization of in vitro assays. In addition, the in vitro method, which gives no benefit, will be directly identified and no longer pursued

Alternatives to animal experiment will dominate the future for ethical and economic reasons. The overall advantage is easily explained: in vitro models are cheaper and have an increased throughput [73]. However, the user will always have to weigh up which test is selected for the purpose including the best significance for predicting human data. For pulmonary development, we suggest a resources- and benefit-driven approach. Resources are all efforts required for the performance of the selected in vitro assay. Benefit includes the prediction of human data and chances for regulatory acceptance, including the status of its validation. Putting this information in a two-dimensional *x*–*y* diagram with *x* = benefit and *y* = resources, we achieve four quadrants from which we can use the first three clockwise for developing orally inhaled drug products (Fig. 2). By passing this way to advanced testing, drug formulation candidates should decrease, and the targeted formulation will be optimized.

Table 2 summarizes in vitro models classified according to their complexity, use of resources and benefits. However, the predictability for in vivo outcomes of the here presented in vitro lung models, especially for predicting the kinetics of absorption and permeability, is still a subject of discussion [74].

**Table 2:** Summary of in vitro assays classified according to their complexity, use of resources and benefit. The examples include only a few of the given possibilities of in vitro tools and even the given validation status and specifications might change in the near future for the described tools

Basic understanding (Less complex assays, low use of resources, medium benefit)								
Assay	Purpose	Throughput	Endpoint	Equipment/material	Specification	Control	Validation status	Ref
pH testing	Avoid tissue damage and coughing	High	pH	pH Electrode	Physiological pH and osmolarity	pH Standards	Accepted	[75]
Osmolality testing	Avoid tissue damage and coughing	High	Osmolality	Osmometer	Physiological pH and osmolarity	Salt solutions		[75]
Aerosol characterization	Estimated deposition in the human lung	Medium	MMAD/GSD/FPF	Cascade impactor	Device dependent	Standard particles with defined sizes		[76]
Screening assays (Less complex assays, low use of resources, significant benefit)								
Assay	Purpose	Throughput	Endpoint	Equipment/material	Specification	Control	Validation status	Ref
Cell viability assay	Estimation of drug/excipients safety	High	Cell viability after 4 h incubation, IC50	MTT-reagent, cell culture equipment, UV/VIS spectroscopy, 96 well plate	2 × 10^5^ cells/mL, A549 (ACC107), Calu-3 (ATCC® HTB 55™), submerged culture conditions, 5% CO_2_ at 37 °C, to passage number 50	HBSS (100% viability) 1% Triton X-100 (0% viability)	Research	[77]
					1.5 × 10^5^ cells/mL, Calu-3 (HTB-55) cells (ATCC), 5% CO2 at 37 °C, to passage number 37–50	5 mM SDS (sodium dodecyl sulphate, 0% viability), buffer (100% viability)	Research	[78]
	Prediction of rat 4 h LC50 values classified according to EPA and GHS	High	Cell viability (IC50), LDH, TEER	MTT/WST-1-reagent, cell culture equipment, UV/VIS spectroscopy, epithelial volt- ohmmeter (EVOM)	EpiAirway™, MucilAir™, A549 (passages 7–23), Calu-3, 3T3 (passages 91–112)	Triton X-100 (0%), 14.7 mg/mL Formaldehyde (0%) Water (100%) Buffer/salt solution (100%)	Research	[79]
Transport Assay	Estimation of bioavailability or transporter activity	High	Permeability coefficient (PAPP)	3 μm 12-well insert, epithelial volt-ohmmeter (EVOM)	Calu-3 cells (passage 32–38),	10 µg/mL sodium fluorescein	Research	[80]
Monocyte inflammation assay	Estimation of safety and efficacy	Medium	TNF/IL-6/IL 10 expression	qPCR instrument, human blood	Monocyte derived macrophages	LPS 100 ng/mL	Research	[81]
Complex assays (High complexity, increased use of resources, significant benefit)								
Assay	Purpose	Throughput	Endpoint	Equipment/material	Specification	Control	Validation status	Ref
Advanced ciprofloxacin testing	Efficacy against *P. aeruginosa*	Low	CFU/mL	CFBE41o- cells, Calu-3, human mucus, 12-well Transwell® plates (0.4 µm)	Calu-3 HTB-55 (ATCC, passage 31–51); TEER > 300 Ω*cm2	Buffer-treatment	Research	[82]
SARS-CoV-2 treatment assay	Estimation of drug efficacy, kinetics viral infection	Medium	Relative viral production, TEER	MucilAir™ HAE, SARS-CoV-2 strain, Vero E6 cells	Nasal HAE: pool 14 donors bronchial HAE 1 donor	Remdesivir	Research	[83]
Tobacco safety assessment	Prediction of adverse effects from tobacco inhalation	Medium	Histological parameters, ciliary beating frequency, cytotoxicity, mRNA expression, secreted mediator concentration	VITROCELL® exposure system, MucilAir™, A549, dTHP cells, EA.hy 926 cells, inverted microscope	MucilAir™ 1 donor separate differentiation THP-1	3R4F cigarette smoke or IQOS MESH Classic Tobacco aerosols	Research	[84, 85]

To increase the predictivity, epithelial cells can be co-cultured or even triple-cultured with immune cells (THP-1, monocyte-derived macrophages (MDM)) and human pulmonary microvascular endothelial cells (HPMEC) to more accurately simulate the air-blood barrier [86–91]. Because the lung is constituted of many cellular and non-cellular (mucus, surfactant) barriers, whose spatial arrangement is essential for a functioning lung physiology, lung organoids were developed. Organoids are defined as 3D in vitro tissue structures simulating a complete in vivo organ [92, 93]. Most organoids are cultivated in a Matrigel matrix and are mostly generated from hPSC (human pluripotent stem cells) [94–97]. The main limitation of organoids is the low standardization of cell cultivation due to the complicated differentiation protocols with countless variations, which causes high costs [98]. Lung-on-a-chip models provide the cell cultivation in a microfluidic device. With a dynamic flow for media transport, a simulation of the function and exposure scenarios of the different lung areas is possible depending on the integrated cell types. However, this technology is still in its infancy [99–102]. A further development of complex in vitro lung systems is the 3D tissue cultures, which are commercially available, for example from MatTek and Epithelix. These systems include ciliated cells, goblet cells with a mucus layer and basal cells in the variant for the upper airways (MucilAir™, Epithelix) or the lower airways (SmallAir™, Epithelix), mucuciliary epithelium on fibroblasts (EpiAirway, MatTek), or the 3D constitution of alveolar epithelial cells, fibroblasts and alveolar endothelial cells (EpiAlveolar™, MatTek) [103–106]. The overall advantage of these ready-to-use systems is the high degree of standardization which is demonstrated by their presence in the ECVAM validation process (EpiAirway™) as mentioned above [72]. Further advantages of these systems are the individual user adaption through various cells (e.g. immune cells), the availability of diseased models and the combination with innovative exposure systems [107]. Primavessy et al. summarized the advantages of commercially available exposure systems which enhance the quality of in vitro systems due to a physiological lung simulation and substance deposition in comparison to static experimental setups [108]. One of the most renowned manufacturers of exposure systems for inhalation toxicology is VITROCELL® Systems. These VITROCELL® devices can expose airborne particles or chemicals (gases, mixtures, NPs) to many formats of cell cultures cultivated in liquid–liquid interface (LLI) or in air–liquid interface (ALI) [109]. In 2019, Kooter et al. investigated asthma patients’ increased sensitivity to copper oxide nanoparticle aerosols using the MucilAir™ (Epithelix) and a VITROCELL® exposure system. They observed a changed response of the model in a diseased state after NP exposure in comparison to the healthy cells by performing a transcriptomic analysis [110]. This experimental setup tries to simulate the particle concentration in the atmosphere more realistically than the calculation of particle concentration in dilution (LLI) [111].

Nevertheless, complex exposure experiments are still limited, due to technical restrictions as to simulating the highest dose of airborne particles and substances compared with surrounding contaminant concentration [112]. Future challenges for the standardization of physiologically relevant exposure systems with ALI cell cultures are the validation of in vitro to in vivo outcomes, the simulation of chronical exposure and the evaluation of the dosimetry (gas versus particles) to identify the exact NOAEL/LOAEL [112, 113].

These challenges were discussed during an international workshop with respiratory toxicology experts in 2018, which evaluated the necessary conditions and setup for an in vitro test system for respiratory safety assessment aiming the ECVAM validation. During the discussion, the main question arose as to which results can be validated to which endpoints regarding, for example, respiratory irritation, sensitization or inflammation. The problem being that no standardization of the established in vitro methods is possible, and, most critically, in vitro models are not compared to human clinical data and only correlated to animal data [68]. Based on these challenges, further research and additional data are still required for the improvement, validation and regulatory acceptance of respiratory in vitro models in order to establish them as alternatives to animal testing and to widen the implementation of the Three Rs principle in pharmaceutical research and industry.

## Data Availability

Data sharing not applicable to this article as no datasets were generated or analysed during the current review article.
